# Suppression of MT5-MMP Reveals Early Modulation of Alzheimer’s Pathogenic Events in Primary Neuronal Cultures of 5xFAD Mice

**DOI:** 10.3390/biom14121645

**Published:** 2024-12-21

**Authors:** Dominika Pilat, Jean-Michel Paumier, Laurence Louis, Christine Manrique, Laura García-González, Delphine Stephan, Anne Bernard, Raphaëlle Pardossi-Piquard, Frédéric Checler, Michel Khrestchatisky, Eric Di Pasquale, Kévin Baranger, Santiago Rivera

**Affiliations:** 1 Inst Neurophysiopathol, CNRS, INP, Aix-Marseille Univ, 13005 Marseille, France; dpilat@mgh.harvard.edu (D.P.); paumierjeanmichel@gmail.com (J.-M.P.); laurence.louis@univ-amu.fr (L.L.); christine.manrique@univ-amu.fr (C.M.); lgarcia@barcelonabeta.org (L.G.-G.); delphine.stephan@univ-amu.fr (D.S.); anne.bernard@univ-amu.fr (A.B.); michel.khrestchatisky@univ-amu.fr (M.K.); eric.di-pasquale@univ-amu.fr (E.D.P.); 2 IPMC, UMR 7275 CNRS-UCA, INSERM U1323, Labex DistAlz, 06560 Valbonne, France; pardossi@ipmc.cnrs.fr (R.P.-P.); frederic.checler@ipmc.cnrs.fr (F.C.)

**Keywords:** matrix metalloproteinase, Alzheimer’s disease, beta amyloid, C99/C83, interleukin-1 beta, neuronal activity, synapse, scavenging systems

## Abstract

We previously reported that membrane-type 5-matrix metalloproteinase (MT5-MMP) deficiency not only reduces pathological hallmarks of Alzheimer’s disease (AD) in 5xFAD (Tg) mice in vivo but also impairs interleukin-1 beta (IL-1β)-mediated neuroinflammation and Aβ production in primary Tg immature neural cell cultures after 11 days in vitro. We now investigate the effect of MT5-MMP on incipient pathogenic pathways that are activated in cortical primary cultures at 21–24 days in vitro (DIV), during which time neurons are organized into a functional mature network. Using wild-type (WT), MT5-MMP^−/−^ (MT5^−/−^), 5xFAD (Tg), and 5xFADxMT5-MMP^−/−^ (TgMT5^−/−^) mice, we generated primary neuronal cultures that were exposed to IL-1β and/or different proteolytic system inhibitors. We assessed neuroinflammation, APP metabolism, synaptic integrity, and electrophysiological properties using biochemical, imaging and whole-cell patch-clamp approaches. The absence of MT5-MMP impaired the IL-1β-mediated induction of inflammatory genes in TgMT5^−/−^ cells compared to Tg cells. Furthermore, the reduced density of dendritic spines in Tg neurons was also prevented in TgMT5^−/−^ neurons. IL-1β caused a strong decrease in the dendritic spine density of WT neurons, which was prevented in MT5^−/−^ neurons. However, the latter exhibited fewer spines than the WT under untreated conditions. The spontaneous rhythmic firing frequency of the network was increased in MT5^−/−^ neurons, but not in TgMT5^−/−^ neurons, and IL-1β increased this parameter only in Tg neurons. In terms of induced somatic excitability, Tg and TgMT5^−/−^ neurons exhibited lower excitability than WT and MT5^−/−^, while IL-1β impaired excitability only in non-AD backgrounds. The synaptic strength of miniature global synaptic currents was equivalent in all genotypes but increased dramatically in WT and MT5^−/−^ neurons after IL-1β. MT5-MMP deficiency decreased endogenous and overexpressed C83 and C99 levels but did not affect Aβ levels. C99 appears to be cleared by several pathways, including γ-secretase, the autophagolysosomal system, and also α-secretase, via its conversion to C83. In summary, this study confirms that MT5-MMP is a pivotal factor affecting not only neuroinflammation and APP metabolism but also synaptogenesis and synaptic activity at early stages of the pathology, and reinforces the relevance of targeting MT5-MMP to fight AD.

## 1. Introduction

Matrix metalloproteinases (MMPs) are a family of multigenic Zn^2+^-dependent endopeptidases with emerging roles in neurodegenerative diseases, such as Alzheimer’s disease (AD) [1,2]. Membrane-type 5 matrix metalloproteinase (MT5-MMP; also known as MMP-24 or η-secretase) is the only MMP primarily expressed in the nervous system [3], where it has been implicated in axonal outgrowth and reorganization following allodynia [4,5] and traumatic brain injury [6]. In physiological settings, MT5-MMP appears to play a role in neural cell differentiation and morphogenesis [7,8]. In addition, MT5-MMP has been suggested to influence synaptic activity by cleaving synaptic proteins alongside γ-secretase [9] or by interacting with AMPA receptor binding protein (ABP) and glutamate receptor interacting protein (GRIP) [10], which are involved in targeting AMPA receptors in the cell membrane. In addition to its role in neuronal plasticity (reviewed in [11]), MT5-MMP has been shown to cleave the amyloid precursor protein (APP) [12,13], a key protein in AD, whose metabolism leads to the accumulation of the APP C-terminal fragment (CTF) (i.e., C99) and its immediate derivative, the amyloid beta peptide (Aβ) [13,14]. The accumulation of Aβ is traditionally considered one of the cornerstones of our understanding of AD [15], but C99 has recently gained ground as an APP fragment whose accumulation can also be harmful, independently of Aβ [16,17,18,19]. It is, therefore, essential to have an in-depth view of the importance of APP metabolites in pathological progression. In line with these idea, we have shown in the transgenic 5xFAD mouse model of AD (Tg) that the absence of MT5-MMP strongly reduces C99 and Aβ levels as well as gliosis/neuroinflammation, and prevents deficits in spatial learning and memory and LTP [13,14]. Furthermore, it has been recently shown that processing of APP by MT5-MMP at the η site results in the production of the η-CTF and contributes to Aβ formation in a β-secretase-dependent manner [20]. Moreover, β- and α-secretase cleavage of η-CTF may also generate N-terminally elongated Aβ species, i.e., Aη-β and Aη-α [21], which impair long-term potentiation (LTP) [22]. Collectively, these data highlight the role of MT5-MMP in AD pathogenesis through its involvement in multiple pathological pathways, particularly those affecting APP metabolism.

There is increasing evidence suggesting that neuroinflammation is a major cause of AD pathogenesis and not just a consequence of brain dyshomeostasis [23,24]. IL-1β is particularly interesting due to its pleiotropic proinflammatory effects in AD [25,26]. In addition, some of the inflammatory effects of IL-1β in the peripheral nervous system (PNS) result from functional interactions with MT5-MMP [27]. We recently showed in primary cultures of relatively immature brain neurons from Tg mice that MT5-MMP deficiency limits basal- and IL-1β- mediated inflammation and hyperexcitability, but does not have a major effect on APP metabolism [28]. The question arises as to how these features, which are important for AD pathology, develop during neuronal maturation and how MT5-MMP affects the underlying mechanisms. To this end, we investigated the effects of MT5-MMP deficiency in mature neuron/astrocyte WT and Tg primary cultures exposed to IL-1β. At 21–24 days in vitro (DIV) these cultures exhibit a dense, active neuronal network with abundant dendritic spines [29,30]. This study sheds new light on the important contributions of MT5-MMP to AD pathogenic mechanisms, including the inflammatory response and neuronal excitability, dendritic spine instability, and the fate of APP CTFs.

## 2. Materials and Methods

### 2.1. Reagents

The inhibitors of γ-secretase DAPT (Tocris, Bio-Techne, Lille, France), α-secretase (GI 254023X (GI hereafter, Tocris, Bio-Techne, Lille, France), and β-secretase C3 (Millipore, Molsheim, France) were used at concentrations of 10, 2, and 10 μM, respectively. The proteasome inhibitor MG132 (MG hereafter, Enzo Life Sciences, Villeurbanne, France) and the autophagolysosome system inhibitor Bafilomycin A1 (BafA1 hereafter, Sigma-Aldrich, Saint-Quentin Fallavier, France) were used at 5 μM and 50 nM, respectively. Proteinase inhibitor was purchased from Calbiochem (Tocris). Poly-L-lysine (#P2636) and RIPA buffer were purchased from Sigma-Aldrich. Recombinant murine IL-1β protein was purchased from PeproTech (Neuilly-sur-Seine, France) and used at 10 ng/mL. All the chemical reagents used in this study were of analytic grade and were purchased from Sigma-Aldrich unless otherwise stated. All the media, fetal bovine serum (FBS), antibiotics, B27, reagents for genotyping, RT-qPCR reagents, and reagents for immunocytochemistry (ICC) were purchased from Thermo Fisher Scientific (Villebon-sur-Yvette, France).

### 2.2. Mixed Neuronal Glial Cultures

All the experimental procedures were conducted in agreement with the authorization for animal experimentation granted by the French Ministry of Research to the Institute of Neuropathophysiology on 9 September 2020 (research project protocol code: APAFIS#23040-2019112708474721 v4) after approval by the National Ethics Committee for Experimental Research (Committee #14). We used WT, MT5^−/−^, Tg, and TgMT5^−/−^ mice from a C57BL6 genetic background [13] and bred Tg and TgMT5^−/−^ male mice with WT and MT5^−/−^ females, respectively. E16 embryos were extracted from the uterine horns of pregnant females anesthetized with pentobarbital (Ceva Santé Animale, Libourne, France) before delivery. Tails were kept at −20 °C until genotyping of the human *PSEN1* transgene was performed [31]. Cerebral cortices were dissected and placed in cold HBSS1X medium and dissociated for 10 min at 37 °C in HBSS1X containing DNAse I (10 µg/mL) and 0.1% trypsin. The enzymatic reaction was then stopped with DMEM solution containing 10% FBS, and mechanical dissociation was further performed through a pipette tip. After centrifugation at 300× *g* for 5 min, 3 × 10^5^ cells/well were plated onto 6-well plates precoated with poly-L-lysine (10 μg/mL), for 2 h in DMEM medium containing 10% FBS and 1% penicillin/streptomycin (P/S). The full medium (1 mL) was further replaced by Neurobasal medium containing B27, 1% glutamine, and 1% P/S for 21 DIV in culture without antimitotic treatment, as previously described [28]. Cells were treated with either IL-1β or the abovementioned inhibitors 24 h before being collected in RIPA buffer for Western blot (WB) analysis or in PBS for RNA extraction using a Nucleospin RNA kit (Macherey-Nagel, Hoerdt, France). In all cases, DMSO was used at the appropriate concentrations under control conditions. For ICC experiments, cells were plated at a 1 × 10^5^ density on 24-well plates on glass coverslips precoated with 500 μg/mL of poly-L-lysine. After 21 DIV, the cells were fixed for 15 min with AntigenFix (Diapath, MM France, Brignais, France). For electrophysiological experiments, the cells were plated as described above for ICC and recorded between 21 and 24 DIV.

### 2.3. Western Blot

Protein concentration was measured using a Bio-Rad *DC*^TM^ protein assay kit (Bio-Rad, Marnes-La-Coquette, France), and 30 μg of protein was loaded and separated on 10 to 15% SDS-PAGE gels or low-molecular weight Tris–Tricine precast gels (Thermo Fisher Scientific), and transferred to nitrocellulose membranes (Dutscher, Brumath, France). After blocking, the membranes were probed with the following antibodies directed against them: MT5-MMP (our own antibody previously described in [13], 1/500), the APP N-terminal fragment (22C11, 1/1000, Millipore), the APP C-terminal fragment (APP-CTF, 1/1000, Sigma-Aldrich), human APP/Aβ (6E10 1/500, Ozyme, Saint-Cyr l’Ecole, France), β-III tubulin (Sigma-Aldrich, 1/1000), N-cadherin (1/1000, BD Biosciences, Le Pont de Claix, France), and β-actin (1/5000, Sigma-Aldrich). Then, the samples were incubated with horseradish peroxidase-conjugated secondary IgG antibodies (Jackson ImmunoResearch, West Grove, PA, USA). Immunoreactive bands were visualized using an enhanced chemiluminescence (ECL) kit (Dutscher) and quantified using Fiji/ImageJ software (NIH), version # 2.14.0/1.54f. Note that WB data are represented in separate columns when the samples were not adjacent in a gel.

### 2.4. Reverse Transcription-Quantitative Polymerase Chain Reaction (RT-qPCR)

Total RNA was extracted from cells cultured for 21 DIV using a Nucleospin RNA Kit (Macherey-Nagel, Hoerdt, France) according to the manufacturer’s recommendations. Single-stranded cDNA was synthesized from 500 ng of RNA using a High-Capacity RNA to cDNA^TM^ kit (Thermo Fisher Scientific) suitable for quantitative PCR. Twenty-five nanograms of cDNA was subjected to qPCR using a Fast Real-Time PCR System (Thermo Fisher Scientific). For each experiment, cDNA samples were analyzed in duplicate, and relative gene expression was obtained using the comparative 2^−(ΔΔCt)^ method after normalization to *Gapdh* (Mm99999915_g1) as a housekeeping gene [28,31]. The expression of the following genes was studied: *Mmp24* (Mm00487721_m1), *Mmp14* (Mm00485054_m1), *hAPP* (Hs00169098_m1), *hPSEN1* (Hs00997789_m1), *App* (Mm01344172_m1), *Il-1β* (Mm01336189_m1), *Ccl2* (Mm00441242_m1), *Il6* (Mm00446190_m1), *Il18* (Mm00434226_m1), *Casp1* (Mm00438023_m1), *Mmp2* (Mm00439498_m1), *Mmp9* (Mm00442991_m1), *Ide* (Mm00473077_m1), *Ace* (Mm00802048_m1), *Ece* (Mm01187091_m1), *Mme* (Mm01285049_m1), *Adam10* (Mm00545742_m1), *Bace1* (Mm00478664_m1), *Psen1* (Mm00501184_m1), and *Psen2* (Mm00448405_m1).

### 2.5. MTT Test

Cell viability was evaluated using the 3-(4,5-dimethylthiazol-2yl)-2,5-diphenyl tetrazolium bromide (MTT) assay (Sigma-Aldrich), which measures mitochondrial activity in living cells. A solution at 5 mg/mL was prepared into Neurobasal medium and added to the cultures at a final concentration of 0.5 mg/mL for 3 h at 37 °C and 5% CO_2_. The medium was removed and replaced with 200 μL of DMSO, 100 μL of which were transferred to a 96-well plate for absorbance (OD) measurements via a spectrophotometer at 550 nm. The data were calculated as the percentage of live cells = (transfected cell OD_550_/control cell OD_550_) × 100. Mean values ± SEM were obtained from at least five animals per genotype.

### 2.6. Viral Infections

We used an empty serotype 10 adenovirus-associated AAV10 (hereafter AAV-empty) or an AAV10 encoding human C99 (hereafter AAV-C99), both of which are under the control of the human synapsin-1 promoter, within a packaging rAAV2-Rh10 plasmid [17]. At 20 DIV, WT and MT5^−/−^ neurons were incubated with 2 μL of these AAVs (5 × 10^12^ vg/mL, MOI = 2.5 × 10^4^) in 1 mL of culture media and treated at 24 DIV with DAPT (10 μM) alone or with BafA1 (50 nM) or MG132 (5 µM) cotreatments, and cell lysates were collected at 25 DIV for WB analysis, as described above. Cells were harvested in RIPA buffer containing proteinase inhibitors, and supernatants were collected to measure Aβ40 levels using ELISA (see below). In addition, lysates from AAV-C99-infected cells were run on the WB as a molecular weight control for C99 and C83.

### 2.7. ELISA

Total levels of human and mouse Aβ38, Aβ40, and Aβ42 in culture supernatants were determined by ELISA using the V-PLEX Plus Aβ Peptide Panel 1 (4G8) Kit (Meso Scale Discovery, Rockville, MD, USA) (Meso Scale Discovery, Rockville, MD, USA). The levels of human Aβ40 in the supernatants of AAV-infected cells were evaluated using an Aβ40 ELISA kit (#KHB3481, Thermo Fisher Scientific). The levels of mouse Aβ40 in culture supernatants after inhibitor treatment were evaluated using an Aβ40 ELISA kit (#KMB3481, Thermo Fisher Scientific). For the detection of IL-1β and MCP-1 in cell supernatants, we used the murine IL-1β and MCP-1 ELISA Development Kits (PeproTech), respectively. All assays were performed according to the manufacturer’s recommendations.

### 2.8. Primary Neuron Transfection with GFP and Analysis of Dendritic Spines

As previously described [31], the pEGFP-N1 (Clontech, Saint-Germain-en-Laye, France) plasmid-encoding GFP was amplified from *E. coli* DH5α (Thermo Fisher Scientific) and purified using the NucleoBond Xtra Midi Plus EF (Macherey-Nagel) according to the manufacturer’s recommendations. At 20 DIV, the neurons were transfected with the GFP-encoding plasmid using lipofectamine 3000 (Thermo Fisher Scientific) according to the manufacturer’s recommendations. Briefly, 1 μg of plasmid was mixed with lipofectamine 3000 reagents and added to the cultures for 2 h. The supernatants were then removed and replaced with conditioned media from control feeder cultures from the same animal. Cells were allowed to express GFP for 24 h before being fixed with AntigenFix (Diapath, MM France, Brignais, France) and blocked with PBS (1X), BSA 3%, and 0.1% Triton X-100 for 1 h (blocking buffer). The GFP signal was amplified with anti-GFP antibodies (1/500, Roche Diagnostics, Meylan, France), and the appropriate Alexa Fluor^®^ 488-conjugated secondary antibody was used at a 1/800 dilution in blocking buffer. Hoechst (0.5 μg/mL) was used to stain the nuclei. Thirty confocal stacks per image were generated, and 3D reconstruction was performed using the surpass module of the Imaris software 9.1. Imaris automatically detected dendrite and spine shapes based on the GFP signal. Spine density was calculated by dividing the total number of spines by the length of the dendrite.

### 2.9. Electrophysiology

#### 2.9.1. Whole-Cell Patch Clamp

Whole-cell recordings of selected pyramidal neurons stimulated or not with IL-1β (10 ng/mL) were performed using an Axopatch200B amplifier (Axon Instruments, Axon Digidata 1550, Molecular Devices, San Jose, CA, USA) under an infrared differential interference contrast microscope Zeiss Examiner A1 (Zeiss Meditech, Marly le Roi, France) coupled to a camera (Jenoptik ProgRes MF; Carl Zeiss, Jena, Germany). Patch microelectrodes (1.5 mm OD, borosilicate filament glass, BF150 from WPI) were pulled using a Narishige PP-830 electrode puller (Fulbourn, Cambridge, UK) and filled with the following solution for current-clamp experiments on the day of the experiment: 140 mM KCl, 10 mM N-2-hydroxyethylpiperazine-N-2-ethanesulfonic acid (HEPES), 10 mM ethylene glycol-bis (b-aminoethylether)-N,N,N’, N-tetraacetic acid (EGTA), 1 mM MgCl_2_, 1 mM CaCl_2_, and 4 mM Mg-ATP/0.4 mM Na_2_-GTP (pH 7.4, balanced with KOH). In voltage-clamp mode, to record the miniature postsynaptic currents (miniz), pipettes were filled with 100 mM CsCl, 30 mM CsFl, 10 mM N-2-hydroxyethylpiperazine-N-2-ethanesulphonic acid (HEPES), 5 mM ethylene glycol-bis (b-aminoethylether)-N,N,N’, N-tetraacetic acid (EGTA), and 1 mM MgCl_2_. On the day of the experiment, 2 mM CaCl_2_, and 4 mM Mg-ATP/0.4 mM Na_2_-GTP (pH 7.4, balanced with CsOH) were added.

Pipettes (4–6 MΩ) were applied to neurons using a Sutter motorized microdrive (ROE200, Sutter Instrument Co WPI, Friedberg, Germany). The offset between the reference electrode and the patch pipette was zeroed upon contact with the extracellular medium of the recording chamber. The artificial cerebrospinal fluid (aCSF) consisted of 140 mM NaCl, 3 mM KCl, 10 mM HEPES, 10 mM glucose, 2.5 mM CaCl_2_, and 1 mM MgCl_2_ (pH 7.4). TTX (300 nM) was added to aCSF to record global miniature postsynaptic currents (gPSCs). The reference electrode was an Ag-AgCl wire connected to the extracellular solution. Resting membrane potential values were not corrected for junction potential. The selected neurons had gigaohm seals (typically 1–5 GΩ) and a stable resting membrane potential. Recordings were obtained in current-clamp mode, and the output bandwidth was set to 10 kHz. The selected neurons had an access resistance < 15 MΩ that was not compensated. The steady-state membrane potential was measured after a hyperpolarizing test pulse of 100 pA/500 ms, the value of which gave the input resistance of the membrane.

#### 2.9.2. Network Spontaneous Activity

The experiments were performed in normal aCSF. Since the neural network in culture is spontaneously active, it is possible to record spontaneous long-lasting depolarizations over which bursts of action potentials (APs) can be observed. The occurrence per minute and the duration of these depolarizations were measured in the presence or absence of APs on top of the depolarization. The duration was measured at the base and the amplitude was measured at the peak of a depolarization.

#### 2.9.3. Induced Firing

The experiments were performed in normal aCSF. Whole-cell current-clamp recordings were filtered at 10 kHz. After the 100 pA/500 ms hyperpolarizing pulse (see above), incremental depolarizing pulses of 40 pA/500 ms elicited repetitive firing in control and IL-1β-treated cultures. The generated excitability curves were compared in terms of the number of APs as a function of the amplitude of the stimulating current.

#### 2.9.4. Action Potential Threshold, Amplitude and Duration

The AP threshold was the average potential measured after obtaining the rheobase current for each cell, i.e., for an AP occurrence of 50% against a stimulating current. The voltage thresholds of 5 individual APs were then averaged. The amplitude of the AP was measured from the voltage threshold to the peak and the duration at half amplitude.

#### 2.9.5. Global Synaptic Currents

In voltage-clamp mode, the cells were held at −50 mV and in TTX-supplemented saline, and miniature global post-synaptic currents (gPSCs) were recorded for 5 min (bandwidth, 1 kHz), after a 5 min recovery period after breaking through the plasma membrane. We did not distinguish between excitatory or inhibitory synaptic currents. The analysis was run offline using Clampfit11 (Axon Instruments, Axon Instruments, Axon Digidata 1550, Molecular Devices, San Jose, CA, USA) routines. Miniature gPSCs were selected individually for each neuron of each genotype and pharmacological condition. Statistics were then obtained on the mean amplitude and mean frequency of miniature gPSCs’ occurrence over 5 min to generate histograms. One way to compare synaptic strength is to determine the synaptic charge transfer (CT). This term includes the amplitude and duration of a postsynaptic current (PSC). Here, the CT was obtained by multiplying the PSC area by the instantaneous frequency.

### 2.10. Statistics

All values represent the mean ± SEM of the number of independent cultures analyzed or the number of recorded neurons, as indicated in the figure legends. For statistical analyses, we used Student’s *t*-test to compare 2 groups or ANOVA followed by the Fisher’s LSD post hoc test to compare more than 2 groups. Two-way ANOVA was used to specifically analyze induced firing. Statistical significance was set at *p* < 0.05. Analyses were performed with the GraphPad Prism version 6.0 software (San Diego, CA, USA).

## 3. Results

### 3.1. MT-MMP and Transgene Expression, and Neuronal Viability

We used mixed cortical neuron/astrocyte cultures at 21 DIV, which exhibit functionally mature synapses with defined dendritic spines and complex electrophysiological activity [29]. As expected, neither the *Mmp24* gene nor the encoded MT5-MMP protein were detected by RT-qPCR or WB in MT5^−/−^ and TgMT5^−/−^ cells compared to their respective WT and Tg controls (Supplementary Figure S1A,B). In addition, IL-1β treatment (10 ng/mL) did not affect the mRNA or protein levels of MT5-MMP in WT or Tg cells (Supplementary Figure S1A,B). We also investigated the expression of MT1-MMP (also known as MMP-14, encoded by the *Mmp14* gene), a close homolog of MT5-MMP sharing proamyloidogenic features [11,31,32]. We found no changes in *Mmp14* mRNA regardless of genotype or treatment (Supplementary Figure S1C).

**Figure 1:**
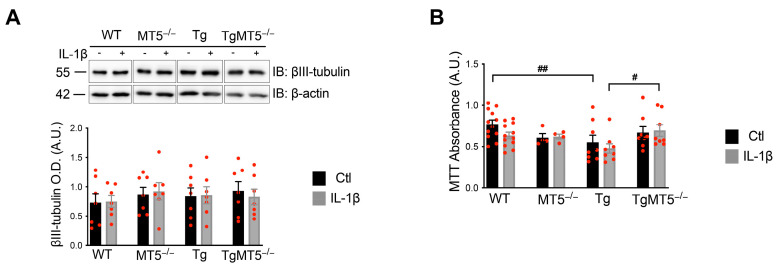
β-III tubulin levels and cell viability. (**A**) Detection of βIII-tubulin levels by WB with the corresponding quantification (bottom panel) normalized to β-actin levels. (**B**) Histogram showing the quantification of cell viability using the MTT assay. Black bars represent control (untreated) conditions, and gray bars represent IL-1β treatment conditions (10 ng/mL for 24 h). Non-adjacent bands in the gels are separated by frames. Values are the mean +/− SEM of 4–12 independent cultures per genotype. ^#^ *p* < 0.05; ^##^ *p* < 0.01 between genotypes. ANOVA followed by post hoc Fisher’s LSD test. IB: immunoblot. OD: optical density. A.U.: arbitrary units. The red dots represent individual values for each experimental condition.

Specific neuronal marker β-III tubulin was unchanged in all experimental conditions (Figure 1A) and the MTT assay revealed a 28% decrease in viable cells in untreated Tg cultures compared to WT, which was not observed in TgMT5^−/−^ cultures (Figure 1B). Upon IL-1β treatment, TgMT5^−/−^ cultures showed 31% higher MTT values than Tg (Figure 1B). Taken together, these data suggest that MT5-MMP deficiency helps to preserve cellular homeostasis in the context of nascent expression of mutant *hAPP* and *hPSEN1* transgenes. Both transgenes were readily detected by RT-qPCR, and no differences were detected between the experimental conditions (Supplementary Figure S2A,B), suggesting that the divergent phenotypes of the Tg and TgMT5^−/−^ cells were not due to differences in the expression of the human transgenes. To complete and complement the *hAPP* and *hPSEN1* transgenes (Supplementary Figure S2A,B), we also measured the mRNA levels of the murine APP (*mApp*) gene (Supplementary Figure S2C). Compared with those in untreated cells, these parameters were altered only in TgMT5^−/−^ cells after IL-1β treatment, in which they were significantly lower (55%) than those in Tg-treated cells treated with IL-1β (Supplementary Figure S2C).

### 3.2. MT5-MMP Deficiency Alters the Expression of Inflammatory Mediators

**Figure 2:**
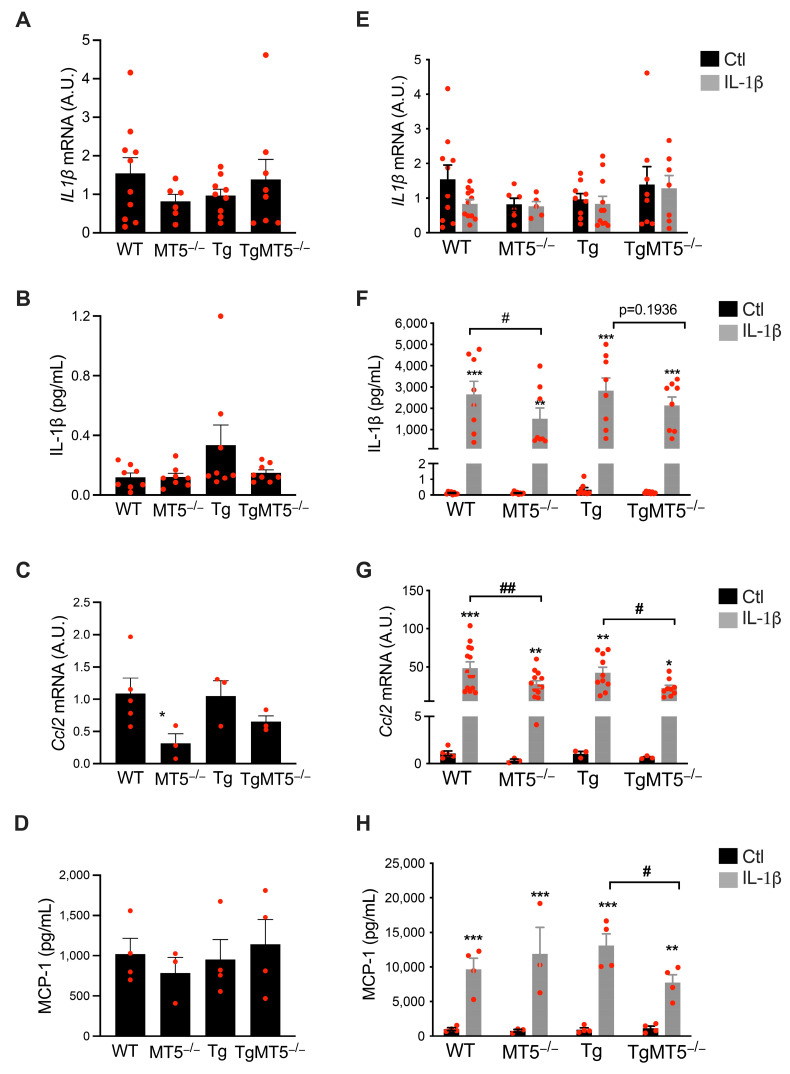
MT5-MMP deficiency selectively alters the expression of inflammatory mediators. IL-1β mRNA (**A**) and protein (**B**) levels and Ccl2/MCP-1 mRNA (**C**) and protein (**D**) levels under untreated conditions. IL-1β mRNA (**E**) and protein (**F**) levels and Ccl2/MCP-1 mRNA (**G**) and protein (**H**) levels in untreated and IL-1β-treated cells, respectively. mRNA levels of *Il1β* and *Ccl2* were analyzed by RT-qPCR. Data values were normalized to *Gapdh*, which was used as a housekeeping gene (**A**,**C**,**E**,**G**). IL-1β and MCP-1 protein levels were measured by ELISA (**B**,**D**,**F**,**H**). Black bars represent control (untreated) conditions and gray bars represent IL-1β-treated conditions (10 ng/mL for 24 h). Values are the mean +/− SEM of 3–14 independent cultures per genotype. * *p* < 0.05, ** *p* < 0.01, and *** *p* < 0.001 between untreated and treated cultures within the same genotype; ^#^ *p* < 0.01 and ^##^ *p* < 0.01 between genotypes. ANOVA followed by post hoc Fisher’s LSD test. A.U.: arbitrary units; ns: non-significant. The red dots represent individual values for each experimental condition.

It is known that MT5-MMP deficiency affects the expression of genes encoding key factors commonly associated with AD. We first evaluated whether the basal levels of two key AD inflammatory mediators were affected. *Il1β* mRNA levels remained unchanged in all genotypes (Figure 2A), whereas protein levels were significantly increased by 260% in Tg cells with respect to WT. This increase was nearly prevented in TgMT5^−/−^, with levels down by 55% compared to Tg (*p* = 0.07) (Figure 2B). The basal expression of *Ccl2,* which encodes monocyte chemoattractant protein-1 (MCP-1), was reduced by 71% in MT5^−/−^ cells and was unchanged in the Tg background (Figure 2C). However, our ELISA results did not follow the same pattern, as MCP-1 levels remained constant in all the genotypes (Figure 2D).

Surprisingly, IL-1β exposure did not significantly regulate its own mRNA (Figure 2E). However, the lack of MT5 downregulated IL-1β levels by 42% in MT5^−/−^ cultures, as demonstrated via ELISA (Figure 2F).

Furthermore, IL-β treatment induced massive upregulation of *Ccl2* in all genotypes, which was consistent with the known regulatory effect of IL-1β on *Ccl2* [33,34], but the absence of MT5 significantly reduced this increase by 43% in the MT5^−/−^ group and by 47% in the TgMT5^−/−^ group (Figure 2G). The induction of mRNA levels was accompanied by a strong increase in MCP-1 in all genotypes, which was 40% lower in TgMT5^−/−^ compared to Tg (Figure 2H).

In a similar fashion to *Ccl2*, the expression of *Il6,* another IL-1β-regulated gene, was strongly upregulated in all genotypes after IL-1β exposure, but this increase was 45% lower in MT5^−/−^ than in WT cells (Supplementary Figure S3a). *Il18* and *Il1*β are both under the control of the NF-κB pathway, where transcriptional activity is supported mainly by *Nfkb1* [35]. *Il18* mRNA levels were unchanged between the WT and MT5^−/−^ groups. Instead, we observed a decrease in TgMT5^−/−^ cells compared to Tg cells in basal conditions, which was even more pronounced after IL-1β treatment (Supplementary Figure S3B). Pro-IL-18 and pro-IL-1β are both converted to their active forms by caspase 1, which is encoded by the *Casp1* gene, through inflammasome formation [36]. Basal *Casp1* expression remained stable in all genotypes. In contrast, IL-1β treatment induced 100% and 157% increases in *Casp1* levels in WT and Tg cells, respectively, which were prevented in MT5^−/−^ and TgMT5^−/−^ cells (Supplementary Figure S3C). Overall, in most cases, MT5-MMP deficiency led to attenuated levels of inflammatory mediators, especially following treatment with IL-1β.

### 3.3. N-Cadherin Is Not Affected by MT5-MMP Deficiency or IL-1β

In contrast to what has been described in the PNS [27], N-cadherin processing by MT5-MMP in response to IL-1β was not affected under our conditions. This is supported by the unaltered levels of full-length N-cadherin in all genotypes (Supplementary Figure S4). This finding is in agreement with our previous work in 11 DIV cultures [28].

### 3.4. Effects of MT5-MMP Deficiency and IL-1β Treatment on Dendrite Spine Integrity

**Figure 3:**
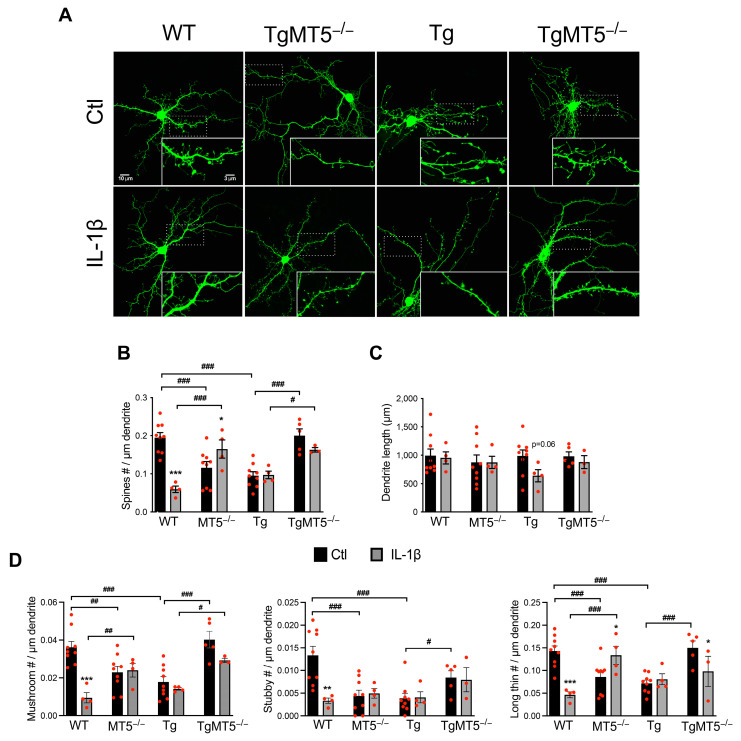
MT5-MMP deficiency and IL-1β modulate the density and shape of dendritic spines. (**A**) Confocal micrographs showing neuronal primary cultures transfected with a plasmid-encoding green fluorescent protein (GFP) and labeled with anti-GFP antibodies to visualize dendritic arborization. Scale bar: 10 μm and 3 µm in the insets. (**B**) Quantification of the number of dendritic spines and (**C**) total dendrite length (μm). (**D**) Quantification of dendritic spines classified by shape: mushroom, stubby, and long thin spines. (**B**–**D**) Data were acquired using the Imaris software. Black bars represent control (untreated) conditions and gray bars represent IL-1β-treated conditions (10 ng/mL for 24 h). The values are the mean +/− SEM of 3–9 independent cultures per genotype. * *p* < 0.05, ** *p* < 0.01, and *** *p* < 0.001 between untreated and treated cultures of the same genotype; ^#^ *p* < 0.01, ^##^ *p* < 0.01, and ^###^ *p* < 0.001 between genotypes. ANOVA followed by post hoc Fisher’s LSD test. The red dots represent individual values for each experimental condition.

Early synaptic dysfunction likely precedes overt pathology and, thus, appears to be a forerunner of late pathological changes. In addition, MT5-MMP [9] and IL-1β [37] have been shown to independently affect neuronal activity, and our previous work demonstrated that, together, they modulate this activity in an immature neuronal network [28]. We thus investigated the effects of genotype and IL-1β treatment on 21–24 DIV cultures, which have a well-developed neuronal network and mature synapses [29]. To this end, we transfected neurons with a GFP plasmid and performed a detailed analysis of dendrites and dendritic spines using Imaris-based 3D reconstruction (Figure 3A). Under untreated conditions, spine density was significantly lower in MT5^−/−^ (42%) and Tg (52%) neurons than in WT neurons, whereas spine density was preserved in TgMT5^−/−^ neurons (Figure 3B). Exposure to IL-1β affects spines in a genotype-dependent manner. The cytokine reduced the spine number by 68% in WT neurons while stimulating a significant recovery in MT5^−/−^ neurons, with values close to those found in untreated WT cells (Figure 3B). Notably, IL-1β did not affect the spine number in Tg or TgMT5^−/−^ cells, indicating the predominant influence of the genotype over that of inflammation (Figure 3A,B). Dendrite length remained stable under all conditions, although a nearly statistically significant decrease in the length of the Tg dendrites exposed to IL-1β was detected (Figure 3C). We then classified spines according to their shape (mushroom, stubby, thin, or filopodia), as spine shape has been associated with distinct synaptic activity (reviewed in [30]). Under basal conditions, the number of mushroom, stubby, and thin spines was significantly lower in MT5^−/−^ and Tg neurons than in WT neurons (Figure 3D), while the number of these spines was preserved in TgMT5^−/−^ neurons. IL-1β treatment reduced the number of mushroom, stubby, and long thin spines on WT neurons. It also reduced the number of mushroom spines on TgMT5^−/−^ neurons but increased the number of long thin spines on MT5^−/−^ neurons. Overall, the absence of MT5-MMP has a dual effect: it appears to compromise spine integrity in WT neurons while helping to prevent potential damaging effects in the AD condition associated with the expression of human transgenes.

### 3.5. Membrane and Spike Properties of Pyramidal Neurons

Table 1 summarizes the intrinsic membrane properties of pyramidal neurons in our primary cultures, as monitored after breaking through the cell membrane in voltage-clamp mode in normal aCSF with K-Glu in the recording pipette (K-Glu lines in Table 1). In untreated cultures, the membrane capacitance (roughly representing the volume of the cell body and proximal branching) ranged from 50 to 66.2 pF, and input resistance ranged from 194.6 to 246.8 MΩ. The only change observed in membrane properties was induced by IL-1β treatment, which reduced input resistance in Tg neurons by 54% compared to WT neurons. When cells were recorded with CsCl (in)/TTX (out) solutions (Table 1, CsCl lines), membrane capacitance and input resistance were not modified in WT, MT5^−/−^, or TgMT5^−/−^ cells. However, IL-1β treatment in Tg cells increased membrane capacitance by 166% and decreased resistance by 61% compared to IL-β treatment in WT cells. These data suggest that the AD genotype or MT5-MMP deficiency had no effect on membrane properties. In contrast, IL-1β affected these parameters, in agreement with the findings of previous work [38,39]. Curiously, in our case, only Tg neurons were affected.

The resting membrane potential (Vrest) was not modified in WT, Tg, or TgMT5^−/−^ neurons, while a significant depolarization at −40 mV was measured in MT5^−/−^ neurons compared to WT (−55.2 mV), but not in TgMT5^−/−^ compared to Tg (Table 1). IL-1β treatment prevented this 15 mV shift in MT5^−/−^ neurons, whereas no significant change in Vrest was observed in the other genotypes. The observed changes may underlie alterations in potassium channels known to regulate Vrest.

Next, we analyzed the properties of single action potentials (APs; Table 1). Under untreated conditions, the membrane potential threshold of single APs (V threshold) was significantly depolarized only in MT5^−/−^ neurons compared to WT neurons. IL-1β treatment did not alter the threshold in MT5^−/−^, Tg, or TgMT5^−/−^ cells. In contrast, IL-1β had a dramatic effect on WT cultures, as only 2 of the 14 recorded neurons showed spontaneous or induced APs. Therefore, the V threshold is marked as not determined (ND) in Table 1. These data are consistent with those in the literature demonstrating a direct effect of IL-1β on ion channels [40].

Spike amplitude in untreated cells, which ranged from 110 to 85 mV, was only significantly lower by 22.5% in MT5^−/−^ neurons than in WT neurons, possibly indicating a reduced number of somatic voltage-dependent sodium channels. However, spike duration was not affected across genotypes. IL-1β treatment did not influence spike amplitude or duration in MT5^−/−^, Tg, or TgMT5^−/−^ cells. As expected, in WT neurons, we were unable to record spike amplitude and duration upon IL-1β treatment, demonstrating a marked negative effect of the cytokine on WT neurons (marked as ND in Table 1). These results suggest that MT5-MMP may be involved in regulating the number or function of voltage-dependent sodium channels responsible for AP amplitude but is unlikely to be involved in the regulation of the potassium channels implicated in AP repolarization.

### 3.6. Effects of MT5-MMP Deficiency and IL-1β on Spontaneous Neuronal Activity

**Figure 4:**
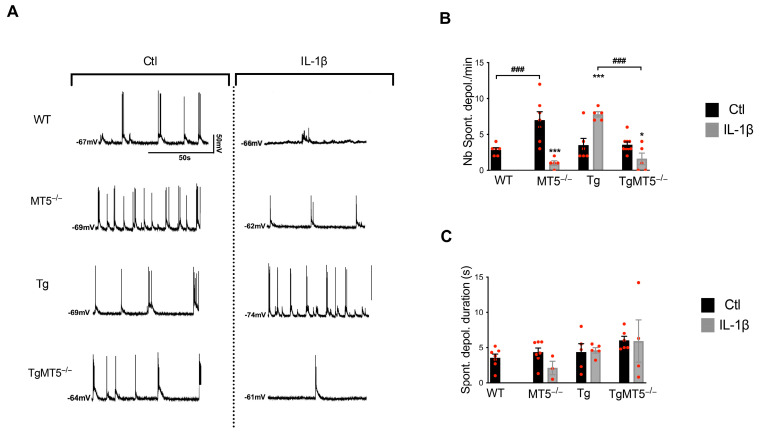
Effect of MT5-MMP deficiency and IL-1β treatment on spontaneous neuronal activity in 21–24 DIV neurons. (**A**) Illustrative traces of spontaneous neuronal activity from visually identified pyramidal neurons in control conditions (left panels) vs. IL-1β-treated cells (right panels). Negative values on the left side of each trace represent the resting membrane potential. (**B**) Histogram representing the number of spontaneous depolarizations observed in 1 min. (**C**) Histogram comparing the duration of individual depolarizations in primary neuronal cultures. Black bars represent control (untreated) conditions and gray bars represent IL-1β-treated conditions (10 ng/mL for 24 h). Note that IL-1β prevented the occurrence of spontaneous AP bursts in 12 out of 15 WT neurons, making it impossible to properly quantify them. Values are the mean +/− SEM of 6, 7, 6, and 9 recorded neurons in untreated conditions from WT, MT5^−/−^, Tg. and TgMT5^−/−^ genotypes, respectively, and 5, 5, and 5 recorded neurons in IL-1β-treated conditions from MT5^−/−^, Tg, and TgMT5^−/−^ genotypes, respectively. * *p* < 0.05 and *** *p* < 0.001 between untreated and treated cultures in the same genotype; ^###^ *p* < 0.001 between genotypes. ANOVA followed by post hoc Fisher’s LSD test. mV: millivolts; s: seconds. The red dots represent individual values for each experimental condition.

Consistent with the hypothesis that synaptic dysfunctions start early in AD [41], we investigated the impact of MT5-MMP deficiency and IL-1β exposure on spontaneous pyramidal cell activity. Representative traces of control and IL-1β-treated cells are shown in Figure 4A. Under basal conditions, the number of spontaneous depolarizations (Figure 4B) reflecting spontaneous network activity was increased by 147% in MT5^−/−^ neurons compared to WT neurons, while no changes were observed in the other genotypes. IL-1β prevented spontaneous AP bursts in 12 out of 15 WT neurons, thus precluding proper quantification (Figure 4B). In contrast, all the untreated WT neurons showed spontaneous depolarization. Remarkably, in MT5^−/−^ neurons, IL-1β prevented the sharp increase in spontaneous depolarizations observed (Figure 4B), but it induced a 120% increase in Tg cells. This increase was abolished in TgMT5^−/−^ neurons, which exhibited 55% fewer spontaneous depolarizations than the untreated controls (Figure 4B).

No changes were observed in the duration of spontaneous depolarization (Figure 4C). Taken together, these findings indicate that functional interactions between MT5-MMP deficiency and IL-1β result in different effects on synaptic activity depending on the WT or Tg genotype. In the latter, MT5-MMP deficiency may contribute to a homeostatic response that prevents an increase in spontaneous bursts and, hence, in neuronal excitability.

### 3.7. Induced Firing Is Altered by MT5-MMP Deficiency, AD Mutations, and IL-1β Exposure

**Figure 5:**
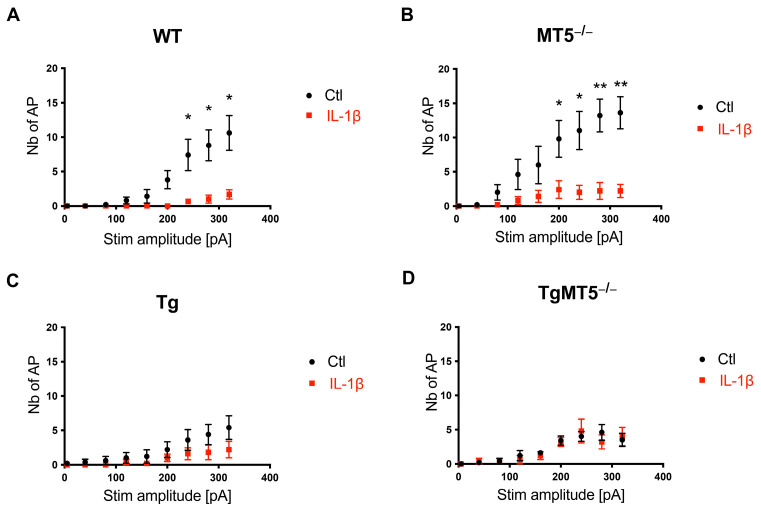
MT5-MMP deficiency and IL-1β modify induced firing. (**A**–**D**) Each point in the plots represents the number of action potentials obtained for each step of depolarization, separately for each genotype (stimulus amplitude: 40 pA increments, during 500 ms). Black dots: control conditions (n = 5, 5, 6, and 5 neurons for each genotype, respectively); red squares: IL-1β treatment (n = 3, 5, 5, and 5 neurons for each genotype, respectively) obtained from at least 3 independent cultures per genotype. Nb of AP: number of action potentials; Stim amplitude (pA): amplitude of the stimulation current inducing the depolarization, in picoamperes. * *p* < 0.05 and ** *p* < 0.01 between untreated and treated cultures of the same genotype. ANOVA followed by post hoc Fisher’s LSD test.

Repetitive firing of APs was measured against an incremented 40 pA/500 ms depolarizing stimulus following a conditioning hyperpolarizing prepulse. Supplementary Figure S5 shows an example of repetitive firing for a WT neuron under untreated conditions. Figure 5A–D shows the relationship between the number of APs and the stimulation current, revealing differences in neuronal excitability between basal and inflamed conditions. Basal somatic excitability was highest in MT5^−/−^ and WT neurons (Figure 5A,B), while Tg and TgMT5^−/−^ neurons were the least excitable (Figure 5C,D).

When comparing spontaneous (Figure 4) and induced excitability (Figure 5), MT5^−/−^ neurons appeared to be hyperexcitable compared to WT neurons, as evidenced by the high number of spontaneous depolarizations shown in Figure 4B. Unexpectedly, the AD background (Tg and TgMT5^−/−^) showed somatic hypoexcitability with no change in network rhythmicity compared to WT (Figure 5C,D). After IL-1β, induced firing was drastically reduced in all genotypes (Figure 5A–D), except in Tg and TgMT5^−/−^ neurons, which already exhibited low excitability in the absence of treatment (Figure 5C,D). Therefore, our results suggest that IL-1β is a master regulator of neuronal excitability in WT neurons, and that the absence of MT5-MMP also influences neuronal excitability. However, this parameter was not affected in the context of the AD background by either IL-1β or MT5-MMP.

### 3.8. Effects of MT5-MMP Deficiency and IL-1β on Global Miniature Synaptic Currents

**Figure 6:**
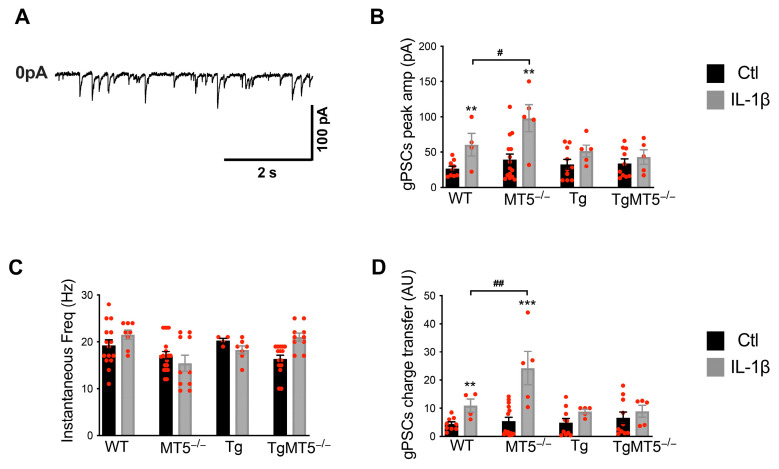
MT5-MMP deficiency and IL-1β affect global miniature synaptic currents. (**A**) Representative trace of global miniature postsynaptic currents (gPSCs) observed in a Tg pyramidal neuron under control conditions. Currents are directed downwards. (**B**) Histogram showing the averaged peak amplitudes of global currents in absolute value, with significant changes observed for WT and MT5^−/−^ neurons under IL-1β conditions. (**C**) Histogram showing the average instantaneous frequency of gPSCs with no significant difference. (**D**) Histogram showing the average charge transfer (CT, indicating the strength of a synapse) of gPSCs. Note that CT is enhanced in WT and MT5^−/−^ neurons under IL-1β conditions. Thus, synapses are strengthened mainly due to the increased amplitude of averaged currents. Black bars: control conditions (n = 9, 15, 10, and 10 neurons for each genotype, respectively); gray bars: IL-1β treatment (n = 4, 5, 5, and 5 neurons for each genotype, respectively). ** *p* < 0.01 and *** *p* < 0.001 between untreated and treated cultures of the same genotype; ^#^ *p* < 0.05 and ^##^ *p* < 0.01 between genotypes. ANOVA followed by post hoc Fisher’s LSD test. pA: picoampere; s: second; Freq: frequency; amp: amplitude; A.U.: arbitrary units. The red dots represent individual values for each experimental condition.

We next sought to relate the observed changes in synaptic integrity, network activity, and somatic excitability to changes in global miniature synaptic activity (gPSCs). We recorded gPSCs in gap-free mode for 5 min, with the voltage clamped at −50 mV. gPSCs were then analyzed offline and individually selected for averaging. A representative trace of gPSCs from a Tg neuron is shown in Figure 6A. Peak amplitude was similar between genotypes under untreated conditions (Figure 6B), whereas IL-1β induced a significant increase in WT (97%) and MT5^−/−^ (149%) cells but had no effect on Tg and TgMT5^−/−^ neurons (Figure 6B). The instantaneous frequency was constant between experimental groups (Figure 6C). Synaptic strength was evaluated via the charge transfer (CT; see methods) (Figure 6D). Basal CT was stable in all genotypes, in contrast to the significant increases induced by IL-1β in WT (148%) and MT5^−/−^ (352%) neurons. IL-1β failed to affect Tg neurons, suggesting that the cytokine strongly affected synaptic activity in a manner restricted to the non-AD context.

Overall, Tg neurons showed no somatic or synaptic hyperexcitability, regardless of TTX presence, IL-1β treatment, or MT5-MMP expression (Figure 5 and Figure 6). However, hyperexcitability in Tg neurons in response to IL-1β treatment occurs through spontaneous network activity (see Figure 4), indicating that hyperactivity is not due to somatic or synaptic hyperfunction in the Tg background. Finally, and of major interest, the absence of MT5-MMP in the WT and Tg genotypes prevented IL-1β-induced hyperexcitability associated with spontaneous network activity.

### 3.9. MT5-MMP Deficiency and IL-1β Treatment Modulate the Expression of Genes Involved in APP/Aβ Metabolism

**Figure 7:**
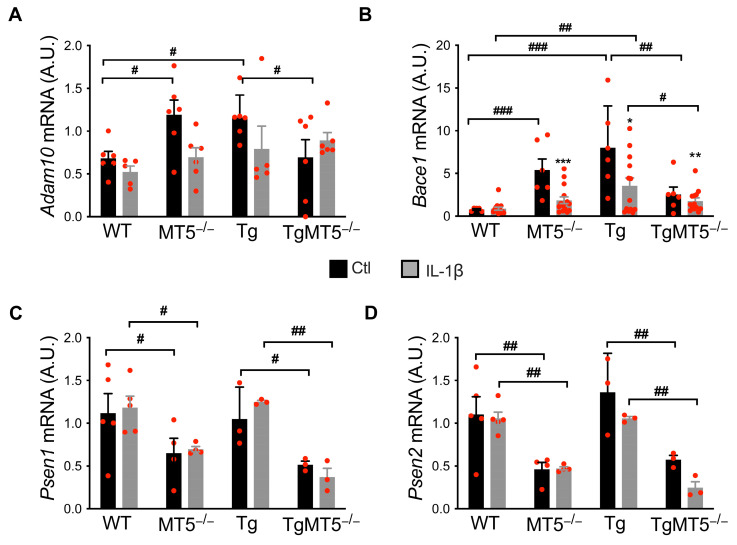
Effects of MT5-MMP deficiency and IL-1β treatment on the expression of genes related to APP metabolism. (**A**–**D**). *Adam10*, *Bace1*, *Psen1*, and *Psen2* mRNA levels were analyzed by RT-qPCR. Data were normalized to *Gapdh*, which was used as a housekeeping gene. Black bars represent control (untreated) conditions, and gray bars represent IL-1β-treated conditions (10 ng/mL for 24 h). Values are the mean +/− SEM of 3–15 independent cultures per genotype. * *p* < 0.05, ** *p* < 0.01 and *** *p* < 0.001 between untreated and treated cultures of the same genotype; ^#^ *p* < 0.01, ^##^ *p* < 0.01, and ^###^ *p* < 0.001 between genotypes. ANOVA followed by post hoc Fisher’s LSD test. A.U.: arbitrary units. The red dots represent individual values for each experimental condition.

We have previously shown that adult TgMT5^−/−^ mice have severely reduced hippocampal and cortical levels of APP CTFs compared to Tg mice [13]. In contrast, MT5-MMP deficiency did not alter APP metabolism in immature primary neurons [28], and we wondered whether this change would occur in the more mature neurons used in the present study. We first examined the expression of genes encoding the canonical APP α-, β-, and γ-secretases. We found similar expression profiles for *Adam10* and *Bace1*, respectively, encoding α- and β-secretases. Compared to WT, basal *Adam10* expression significantly increased by 70% and 66% in MT5^−/−^ and Tg cells, respectively. However, such an increase was prevented in TgMT5^−/−^ cells, whose expression decreased by 40% compared to Tg (Figure 7A). Exposure to IL-1β reduced *Adam10* expression by 42% in MT5^−/−^ cells only (Figure 7A). Likewise, basal *Bace1* expression was upregulated by 700% and 1000% in MT5^−/−^ and Tg, respectively, compared to that in WT. Again, this increase was prevented in TgMT5^−/−^, which showed 68% lower levels compared to Tg (Figure 7B). Interestingly, IL-1β caused a decrease in *Bace1* expression in MT5^−/−^ (66%) and Tg (56%) cells compared to untreated controls. Notably, *Bace1* mRNA levels were significantly reduced by 49% in TgMT5^−/−^ compared to Tg after IL-1β (Figure 7B), while Tg was increased by 400% compared to WT after IL-1β treatment (Figure 7B). Importantly, MT5-MMP deficiency dramatically decreased the basal mRNA levels of the γ-secretase catalytic units *Psen1* and *Psen2* in both AD and non-AD backgrounds (Figure 7C,D). IL-1β did not affect *Psen1* expression, but it reduced that of *Psen2* by 67% in TgMT5^−/−^ compared with the untreated control. In addition, *Psen2* levels in TgMT5^−/−^ were reduced by 58% relative to those in Tg in the untreated groups and by 77% after exposure to IL-1β (Figure 7D). Overall, MT5-MMP deficiency appeared to have a major influence on the expression of canonical secretases.

The expression of other proteinases involved in the degradation of Aβ is summarized in Supplementary Figure S6. We first studied genes encoding MMP-2 and MMP-9, also known as gelatinases A and B, respectively. TgMT5^−/−^ cells expressed 66% less *Mmp2* mRNA than Tg cells, and IL-1β induced a significant 75% decrease in *Mmp2* expression in Tg cells. Basal *Mmp9* mRNA levels remained unchanged in all genotypes. Only MT5^−/−^ cells expressed 44% less *Mmp9* after IL-1β treatment. Other Aβ-degrading metalloproteinases were also examined, namely *Ace*, *Ece*, *Ide*, and *Mme,* which, respectively, encode angiotensin-converting enzyme (ACE), endothelin-converting enzyme (ECE), insulin-degrading enzyme (IDE), and neprilysin (NEP). We found no differences across groups or conditions for *Ace*, *Ece*, and *Ide*. In contrast, basal *Mme* mRNA levels increased by 242% in Tg compared to WT cells and by 306% after IL-1β treatment. MT5-MMP deficiency did not alter this pattern in TgMT5^−/−^ cells; however, in both Tg and TgMT5^−/−^ cells, IL-1β significantly decreased *Mme* levels (Supplementary Figure S6).

### 3.10. Effects of MT5-MMP Deficiency and Secretase Inhibitors on APP Metabolism

**Figure 8:**
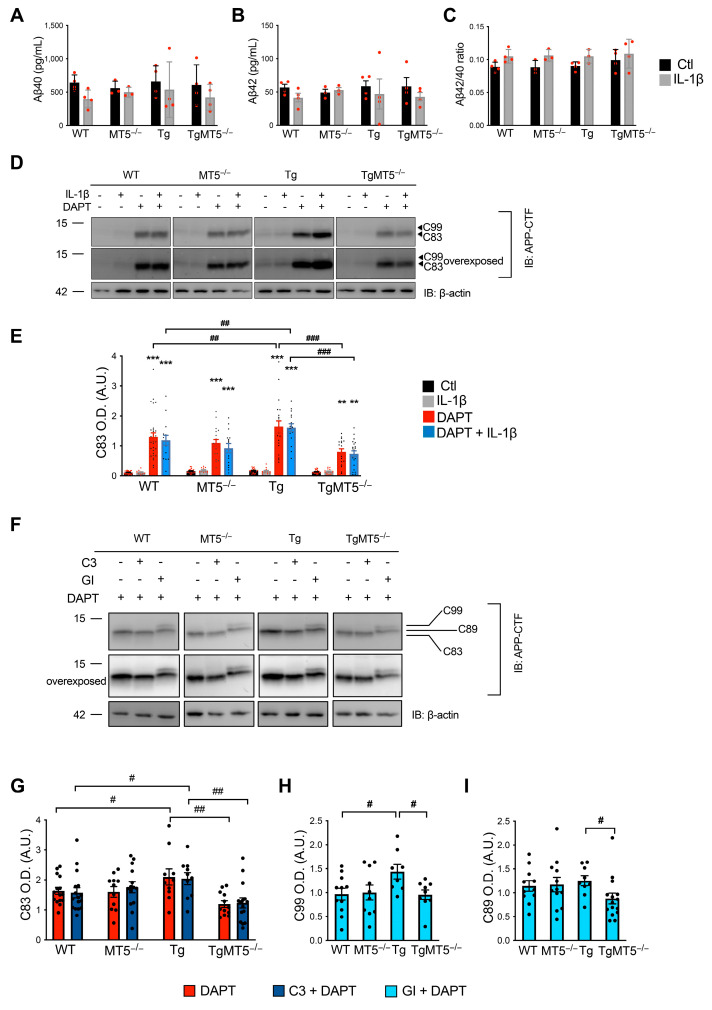
MT5-MMP deficiency does not affect Aβ levels, but it reduces the content of APP CTFs. (**A**–**C**) Measurement of Aβ40 and Aβ42 levels, and the Aβ42/Aβ40 ratio in primary neural cell cultures treated or not treated with IL-1β (10 ng/mL) using the MSD multiplex assay. (**D**) WB analysis of the C83 fragment in primary cultures treated or not treated with IL-1β (10 ng/mL) or DAPT (10 μM) detected by an APP-CTF antibody. (**E**) The corresponding β-actin normalized quantifications. (**F**). WB analysis of APP-CTFs in primary neural cell cultures treated or not treated with DAPT (10 μM) GI (2 μM) and C3 (10 μM) as detected by an APP-CTF antibody. Neurons transduced with AAV-C99 and treated with DAPT were used as positive controls. (**G**–**I**) The corresponding β-actin normalized quantifications of C83, C99, and C89. Non-adjacent bands in the gels are separated by frames. Values for (**A**–**C**) are the mean +/− SEM of 4 independent cultures per genotype; for (**D**,**E**), 15–20 independent cultures per genotype were used, and for (**F**–**I**), at least 9–15 independent cultures per genotype were used. ** *p* < 0.01 and *** *p* < 0.001 between untreated and treated cultures of the same genotype; ^#^ *p* < 0.05, ^##^ *p* < 0.01, and ^###^ *p* < 0.001 between genotypes. ANOVA followed by post hoc Fisher’s LSD test. IB: immunoblot; OD.: optical density; A.U.: arbitrary units. The red and black dots represent individual values for each experimental condition.

The levels and ratios of Aβ40 and Aβ42 were stable, regardless of genotype or IL-1β treatment (Figure 8A–C), and Aβ38 was not detected under our experimental conditions. The levels of full-length canonical APP (APPfl) in both cell lysates and supernatants (sAPP) were unchanged under all experimental conditions, as shown by Western blot analysis in Supplementary Figure S7A,B using the 22C11 antibody against an N-terminal APP epitope. Seemingly, secretase inhibitors did not affect APPfl or sAPP (Supplementary Figure S7C,D). DAPT was primarily used to enable the detection of APP CTFs by the APP-CTF antibody, which recognizes an epitope in the C-terminal region of APP. Under these conditions, we detected an immunoreactive band of the expected size for C83 (Figure 8D), but virtually no signal from the higher molecular weight CTF, C99. C83 levels in Tg cells were significantly higher (30%) than those in WT (Figure 8D,E), and this accumulation was prevented in TgMT5^−/−^ cells, in which a 52% reduction was observed compared with Tg cells. Notably, IL-1β had no significant effect on C83 or C99 levels, regardless of genotype. Taken together, these results suggest that MT5-MMP deficiency modulates APP metabolism in mature primary neurons mainly by limiting the excess of CTFs in the AD context, without affecting Aβ levels.

The preceding data raise the possibility that MT5-MMP deficiency interferes with two major APP secretases, namely β-secretase (BACE-1) and α-secretase, prior to cleavage by γ-secretase. To evaluate this possibility, we treated our cultures with C3 or GI, two well-known inhibitors of β- and α-secretase, respectively. In these conditions, APPfl and sAPP levels remained stable across the experimental groups (Supplementary Figure S7D–F). Contrary to DAPT, C3 or GI alone did not allow the detection of CTFs (not shown). The combination of DAPT and GI abolished the C83 signal and enabled the recovery of C99, suggesting that C99 is primarily converted to C83 by α-secretase (Figure 8F–H). The combination of DAPT and C3 had no effect on C83 compared to DAPT alone (Figure 8F,G). DAPT and GI cotreatment also revealed an intermediate CTF that could represent C89 (Figure 8F,I). Importantly, MT5-MMP deficiency in the Tg background caused a significant decrease in C83 (30%) (Figure 8F,G), C99 (44%) (Figure 8F,H) and C89 (29.8%) (Figure 8F,I) levels compared to those in Tg, demonstrating the ability of MT5-MMP to modulate all CTFs in a β- and α-secretase-independent manner.

**Figure 9:**
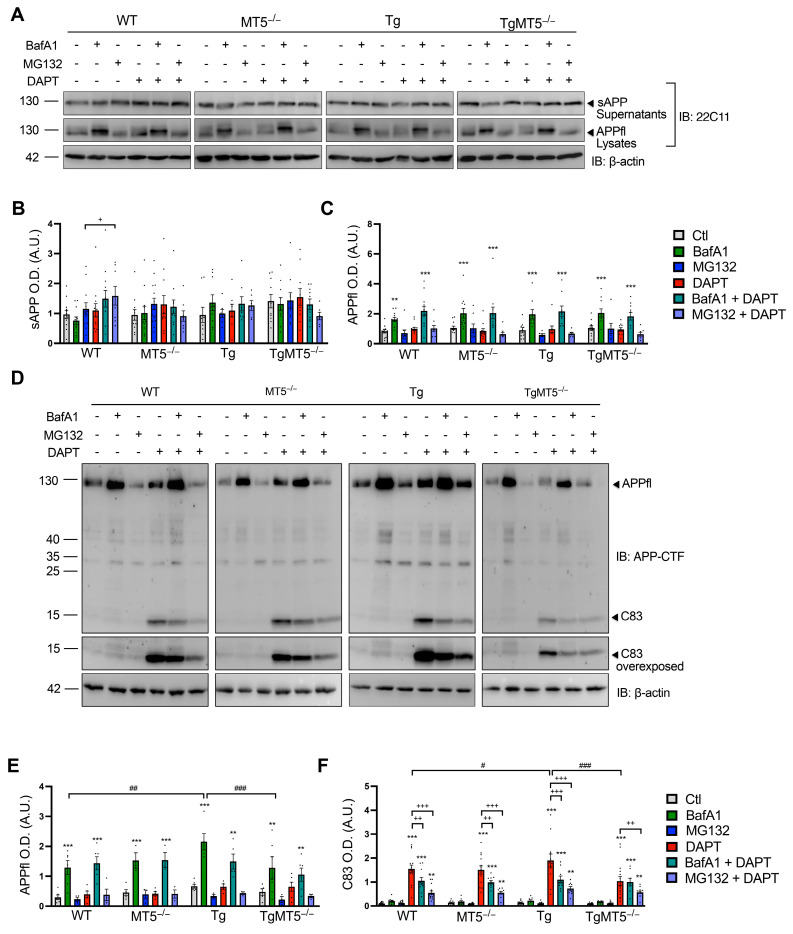
Effects of MT5-MMP deficiency and secretase inhibitors on APP full-length and APP-CTF metabolism. (**A**) WB showing soluble APP (sAPP) and full-length canonical APP (APPfl) levels detected with the 22C11 antibody in primary neural cell cultures treated or not treated with DAPT (10 μM), BafA1 (50 nM), or MG132 (5 μM), and the corresponding β-actin-normalized quantifications for sAPP (**B**) and APPfl (**C**). (**D**) WB showing APPfl and C83 detected with the APP-CTF antibody in primary neural cell cultures treated or not treated with DAPT (10 μM), BafA1 (50 nM), or MG132 (5 μM), and the corresponding β-actin normalized quantifications for APPfl (**E**) and C83 (**F**). Non-adjacent bands in the gels are separated by frames. Graph values are the mean +/− SEM of 8–17 independent cultures (**B**), 6–12 (**C**), 6–14 (**E**), and 3–6 (**F**). ** *p* < 0.01 and *** *p* < 0.001 between untreated and treated cultures of the same genotype; ^#^ *p* < 0.05, ^##^ *p* < 0.01, and ^###^ *p* < 0.001 between genotypes; ^+^ *p* < 0.05, ^++^ *p* < 0.01, and ^+++^ *p* < 0.001 between DAPT and cotreatments of the same genotype. ANOVA followed by post hoc Fisher’s LSD test. IB: immunoblot; OD.: optical density; A.U.: arbitrary units. The black dots represent individual values for each experimental condition.

The above experiments demonstrated the efficacy of γ-secretase in removing C83, as illustrated by the restoration of C83 levels by DAPT (Figure 8D,E). We next asked whether other scavenging systems contribute to C83 degradation. To this end, we used bafilomycin A1 (BafA1) and MG132, which, respectively, inhibit the endolysosomal and proteasomal systems, and first analyzed the effect on sAPP and APPfl levels (Figure 9A–C). The combination of BafA1 and DAPT increased sAPP levels only in WT cultures compared to those treated with BafA1 alone (Figure 9A,B). BafA1 drastically increased APPfl levels in cell lysates from all genotypes, independent of DAPT treatment (Figure 9A,C). In contrast, MG132 had no effect on sAPP and APPfl levels (Figure 9A,C), suggesting that APP was mainly degraded via the endolysosomal pathway. The incubation with the APP-CTF antibody confirmed the recovery of APPfl levels induced by BafA1 in a DAPT-independent manner and the absence of MG132’s effect (Figure 9D,E). In contrast to the 22C11 antibody, APP-CTF antibody immunoreactivity revealed a significant 66% increase in APPfl levels by BafA1 in Tg cells compared to WT cells. This increase was prevented in TgMT5^−/−^ cells (Figure 9D,E). BafA1 and MG132 failed to restore C83 levels (Figure 9D,F). Only DAPT increased C83 content, which peaked at a level 20% greater in Tg than in WT cells (Figure 9D,F) and decreased C83 levels by 55% in TgMT5^−/−^ cells. The low levels of C83 in TgMT5^−/−^ cultures under DAPT were not further modified by BafA1 cotreatment, in clear contrast to the reductions observed in the other groups: WT (33%), MT5^−/−^ (34%), and Tg (43%). Cotreatment with DAPT and MG132 strongly reduced C83 levels in all genotypes compared to those in the respective DAPT controls, although the reduction was less pronounced in TgMT5^−/−^ cells (Figure 9D,F).

### 3.11. Effects of MT5-MMP Deficiency on C99 Overexpression

**Figure 10:**
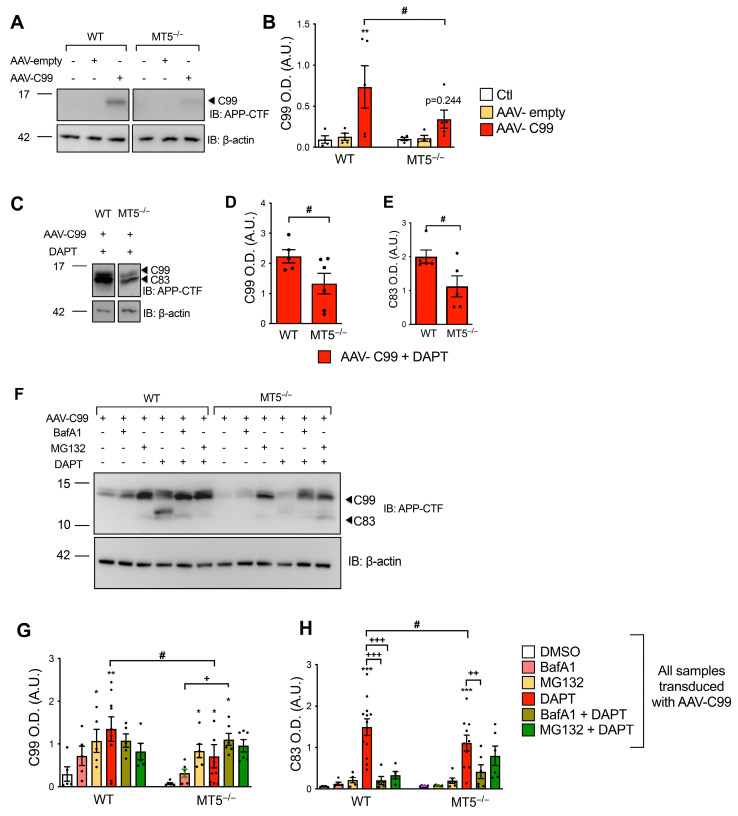
Effects of MT5-MMP deficiency on C99 overexpression in primary neurons. (**A**) WB analysis of C99 levels after transduction with AAV-empty or AAV-C99 (2 μL at 5 × 10^12^ vg/mL) vectors in WT and MT5^−/−^ cells detected with APP-CTF antibodies. (**B**) The corresponding β-actin-normalized quantifications. (**C**) WB analysis of C99 and C83 detected with APP-CTF antibodies in WT and MT5^−/−^ cells after transduction with AAV-C99 (2 μL at 5 × 10^12^ vg/mL), treated or not treated with DAPT (10 μM) and the corresponding β-actin-normalized quantifications for C99 (**D**) and C83 (**E**). (**F**) WB analysis of C99 and C83 detected with APP-CTF antibodies in WT and MT5^−/−^ cells after transduction with AAV-C99 (2 μL at 5 × 10^12^ vg/mL), treated or not treated with DAPT (10 μM) and/or BafA1 (50 nM) or MG132 (5 μM) and the corresponding β-actin-normalized quantifications for C99 (**G**) and C83 (**H**). Non-adjacent bands in the gels are separated by frames. Values for A-H are the mean +/− SEM of 5–12 independent cultures per genotype. * *p* < 0.05, ** *p* < 0.01, and *** *p* < 0.001 between untreated and treated cultures of the same genotype; ^#^ *p* < 0.05 between genotypes; ^+^ *p* < 0.01, ^++^ *p* < 0.01, and ^+++^ *p* < 0.005 between DAPT and cotreatments in the same genotype. ANOVA followed by post hoc Fisher’s LSD test. For (**D**). ^#^ *p* < 0.05, Student’s *t*-test. IB: immunoblot; OD.: optical density; A.U.: arbitrary units; ns: non-significant. The black dots represent individual values for each experimental condition.

The data above indicate that MT5-MMP affects the formation/fate of C83 and C99 in 21 DIV neurons, which can readily remove the excess of CTFs under physiological conditions. To assess how MT5-MMP might interfere with the accumulation of C99 characteristic of AD, we adopted an adeno-associated virus (AAV) infection strategy. We used a C99-encoding AAV (AAV-C99) and an empty (control) AAV [17]. In contrast to cells infected with AAV-empty, cells infected with AAV-C99 readily expressed C99 in the absence of DAPT (Figure 10A). DAPT treatment dramatically increased C99 and C83 levels, whereas GI treatment simultaneously prevented C83 formation and increased C99 levels, confirming that C83 was generated mainly from overexpressed C99 (Supplementary Figure S8).

Once validated, AAV-C99 infection of WT and MT5^−/−^ cells resulted in a significant 52% decrease in C99 levels in MT5^−/−^ cells compared with WT cells (Figure 10A,B). Moreover, there were no significant differences between the AAV-empty and AAV-C99 conditions in MT5^−/−^ cells, supporting the strong influence of MT5-MMP deficiency on C99 fate (Figure 10A,B). DAPT treatment increased C99 and C83 accumulation in WT cells (Figure 10C), but the levels of both CTFs were significantly reduced in MT5^−/−^ cells by 40% for C99 (Figure 10C,D) and 43% for C83 (Figure 10C,E). These results suggest that MT5-MMP helps stabilize C99/C83, while MT5-MMP deficiency promotes C99/C83 clearance. Altogether, these observations in mature cortical neurons are consistent with the in vivo data we obtained from the brains of adult 5xFAD mice [13,14].

To better understand the mechanisms involved in controlling the fate of CTFs after AAV-C99 transduction, we also used BafA1 and MG132 (Figure 10F–H). Compared to the control group, DMSO, MG132, and DAPT, but not BafA1, restored C99 levels in both WT and MT5^−/−^ cells (Figure 10F,G). While no significant differences were observed between WT and MT5^−/−^ cells after BafA1 or MG132 treatment, DAPT decreased C99 levels in MT5^−/−^ cells by 48% compared to those in WT cells. Cotreatment with DAPT and BafA1 did not further modulate C99 levels across genotypes (Figure 10F,G). However, the combination of DAPT and MG132 seemed to interfere with the restoration of C99 in WT cells, as cotreatment resulted in non-significant differences compared to the DMSO control (Figure 10F,G). In contrast, DAPT and MG132 cotreatment significantly restored C99 levels in MT5^−/−^ cells (Figure 10F,G). C83 was undetectable under DMSO and BafA1 conditions and was hardly detectable after MG132 treatment in both WT and MT5^−/−^ cells. On the contrary, DAPT efficiently increased C83 levels in WT and MT5^−/−^ cells, although the levels were significantly lower in MT5^−/−^ cells (27%) (Figure 10F,H). BafA1 prevented the restoration of C83 levels by DAPT in WT and MT5^−/−^ cells, whereas MG132 interfered with the effect of DAPT only in WT but not in MT5^−/−^ cells (Figure 10F,H). Collectively, these data suggest that the fate of C99 and C83 is specifically modulated by different scavenging systems and that MT5-MMP deficiency facilitates the clearance of both CTFs when they are abundantly produced.

## 4. Discussion

The present study confirms that early modulation of MT5-MMP in mature mouse primary cortical neurons helps control pathological events triggered by human *APP* and *PSEN1* genes harboring familial AD mutations. MT5-MMP deletion not only reduced exacerbated neuroinflammation and APP metabolism, but also prevented the loss of dendritic spines in Tg cells. MT5-MMP deficiency also reduced spine density in the WT background, suggesting a synaptogenic role for this MMP under physiological conditions. Changes in spine density were accompanied by a decrease in neuronal excitability promoted by IL-1β in not only WT neurons but also in TgMT5^−/−^ neurons, reinforcing the idea that MT5-MMP deletion is beneficial in the context of AD.

IL-1β treatment had little effect on APP metabolism. In contrast, MT5-MMP deficiency consistently resulted in decreased levels of C99/C83, whether produced endogenously or after AAV-mediated neuronal expression of C99. These findings in primary cortical cells confirm our seminal findings in the brains of adult 5xFAD mice deficient in MT5-MMP. It is noteworthy that C99 was highly labile in our cultures, mainly due to its degradation by α-/γ-secretases and, to a lesser extent, by the proteasome. In contrast, degradation of C83 and canonical APP is primarily driven by γ-secretase and the lysosomal system, respectively. Overall, MT5-MMP deficiency in our cultures revealed dysfunctions related to neuroinflammation, APP metabolism, and synaptic activity that may underlie the pathological features of AD.

### 4.1. MT5-MMP Deficiency Affects IL-1β Signaling Independently of APP Metabolism in Mature Primary Neural Cells

IL-1β is a paradigmatic marker of inflammation in AD [42,43] and is expressed at a 2-fold increase in mRNA levels in 2-month-old 5xFAD mice at the beginning of Aβ accumulation [31]. In the present study, *Il1*β mRNA levels were surprisingly lower in Tg and MT5^−/−^ cells than in cells of the other genotypes. However, as in immature 11 DIV cultures [28], MT5-MMP deficiency seems to prevent the excess of IL-1β generated in basal conditions by Tg cells. The complex interaction between MT5-MMP and IL-1β is also illustrated by the inhibition of IL-1β-mediated induction of *Ccl2* and its MCP-1 encoded protein in TgMT5^−/−^ cells, which paralleled that observed for *Il6* expression in MT5^−/−^ cells and *Casp1* in both MT5^−/−^ and TgMT5^−/−^ cells. Despite the diversity of effects observed depending on genotype/treatment, MT5-MMP deficiency most often affects the inflammatory response, which is likely independent of Aβ levels, as these remain stable under all conditions. The decreased ability of IL-1β to promote neuroinflammation in MT5-MMP-deficient neural cells is in pace with previous observations in the PNS [27]. In this study, MT5-MMP deficiency prevented the inflammatory response to IL-1β in sensory neurons due to impaired cleavage of N-cadherin, a known substrate of MT5-MMP. Consistent with previous data on immature neurons [28], we found no alterations in N-cadherin cleavage in MT5-MMP-deficient cultures, which could explain the reduced response to IL-1β. Alternatively, it has been proposed that IL-1R1, the major IL-1β receptor, undergoes sequential proteolytic processing by the metalloproteinase ADAM10 and by γ-secretase, which is required to elicit IL-1β signaling upon binding to its receptor [44,45]. Type I proteins often share a two-step processing pathway that is driven sequentially by a membrane-bound metalloproteinase and an intramembrane γ-secretase. We hypothesize that MT5-MMP may be involved in this process through cleavage of the extracellular portion of IL-1R1, as it is the case for APP or N-cadherin [12,13,20].

### 4.2. MT5-MMP Deficiency and IL-1β Affect Dendritic Spine Integrity

We report for the first time a drastic reduction in dendritic spine density in primary 5xFAD cortical neurons at 21 DIV, mirroring the reduction in cortical spines at 19 DIV in Tg2576 mice, which also express human APP with the Swedish mutation [46]. This finding suggests a detrimental effect of familial AD mutations on early neurodevelopment under conditions of nascent human transgene expression. In this context, spine loss was markedly prevented in TgMT5^−/−^ neurons, demonstrating the protective effect of MT5-MMP deficiency. However, MT5^−/−^ neurons also exhibited reduced spine density, suggesting a physiological synaptogenic role for MT5-MMP, consistent with its prominent expression early in development [3]. Taken together, these data may reveal opposing effects of MT5-MMP deficiency in AD and physiological contexts. Previous in vitro studies have shown that IL-1β decreases spine density in WT neurons [47,48] through sequential activation of NMDA glutamate receptors, increased intracellular Ca^2+^ influx, and proteasomal degradation of synaptic proteins [49,50]. However, whether MT5-MMP influences glutamatergic activity in AD neurons remains to be determined, especially considering that MT5-MMP binds to AMPA receptor-binding protein (ABP) and glutamate receptor-interacting protein (GRIP), which regulate synaptic targeting of AMPA receptors [10], the latter being major modulators of synaptic function [51].

### 4.3. Impact of MT5-MMP Deficiency and IL-1β on Spontaneous Neuronal Activity

The molecular and structural changes reported above led us to question the possible correspondence of these alterations with changes in neuronal activity, the latter reflecting the functional state of neurons in terms of excitability [52]. IL-1β reduced the number of spontaneous bursts in WT neurons, consistent with previous work correlating a decrease in spontaneous synaptic activity with a decrease in spine number [53], and with the fact that IL-1β alters LTP [54,55] and promotes excitotoxicity [56,57,58]. Notably, the absence of MT5-MMP in TgMT5^−/−^ cells prevented the alterations in spontaneous activity and spine density induced by IL-1β, further suggesting that MT5-MMP deficiency is synaptoprotective in the context of AD. However, the correlation between spine number and neuronal function is lost in MT5^−/−^ cells, as higher spontaneous activity was associated with reduced spine density, implying that other mechanisms are at play. MT5-MMP deficiency under physiological conditions has been associated with morphological changes in iPS-derived human neural cells that could lead to alterations in synaptic function [8]. The observation that MT5-MMP deficiency is associated with a reduction in resting membrane potential only in WT neurons suggests a possible physiological role for MT5-MMP in stabilizing the leaky potassium channels that control Vrest and provides further support for the existence of genotype-specific determinants. In this context, canonical α-, β-, and γ-secretases, which regulate the electrical properties of voltage-gated potassium channels, have been shown to also regulate the proteolytic processing of the channel subfamily members KCNE1 and KCNE2 [59].

Spontaneous network activity did not correspond to the strength of synaptic activity represented by the synaptic charge transfer. This could be because global synaptic excitatory and inhibitory events were recorded indiscriminately, which could mask potential differences between spontaneous current-clamp activity and voltage-clamped synaptic currents. On the other hand, the observed 15 mV depolarizing shift in the MT5^−/−^ resting potential was sufficient to facilitate and increase the rate of spontaneous depolarization without increasing synaptic charge transfer. In terms of somatically induced excitability, MT5^−/−^ neurons were the most excitable neurons, whereas Tg neurons were the least excitable. These data contrast with those obtained from more immature Tg neurons at 11 DIV, where basal hyperexcitability was prevented in the absence of MT5-MMP [28]. Our in vitro data are reminiscent of the early hyperactivity observed in hippocampal neurons from 1-month-old Tg APP/PS1 mice, which shifted to hypoactivity at 3 months of age, probably indicating an early hyperexcitable effect of Aβ and the establishment of adaptative mechanisms as the network matures [60]. The relative stability of AP duration suggested that the afterhyperpolarization spike (AHP) may be altered in the context of somatic excitability. Further experiments are still needed to confirm this point and to identify the channels involved.

The large differences in threshold potential and peak amplitude observed between WT and MT5^−/−^ neurons point to a possible dysregulation of voltage-gated transmembrane sodium channels. In support of this possibility, it has been shown that a MT5-MMP homolog, MMP-9, cleaves 3 different NaV channel isoforms in vitro, and that defective cleavage of mutant Nav channels correlates with the absence of the pain phenotype in some human populations [61].

Overall, the absence of MT5-MMP in WT neurons increases their spontaneous rhythmicity, which is significantly reduced by IL-1β treatment. In the context of AD, IL-1β-induced hyperexcitability is prevented in the absence of MT5-MMP. This finding is consistent with the induction of repetitive firing observed in the WT and MT5^−/−^ vs. Tg and TgMT5^−/−^ neurons. Conversely, the absence of MT5-MMP strongly increased synaptic strength in IL-1β-treated neurons, probably by increasing the amplitude but not the frequency.

### 4.4. MT5-MMP Influences the Formation and Fate of APP CTFs

In contrast to more immature cells (11 DIV), the absence of MT5-MMP influenced APP CTFs in a more mature network (21 DIV). In the presence of γ-secretase inhibition, only C83 was detected, consistent with this CTF being a preferred substrate of γ-secretase [62]. C99 was detected only after combined inhibition of γ- and α-secretase, implying that this CTF is cleaved primarily by α-secretase to produce C83 and, to a lesser extent, by γ-secretase to produce Aβ. These findings support previous work demonstrating not only the processing of C99 by γ-secretase, but also by α-secretase to, respectively, generate Aβ and p3 peptides at the same time as C83 [63]. Moreover, these findings are consistent with the reported localization of α-secretase to the trans-Golgi apparatus and its ability to convert C99 to C83 [64]. Most interestingly, in this experimental setting, MT5-MMP deficiency reduced C99 formation, as is the case in the brains of adult 5xFAD mice [13,14], suggesting that the inhibition of C99 accumulation observed in adult TgMT5^−/−^ mice [13] may in fact begin early in development.

Experiments with BafA1 showed that APP is mainly degraded in the autophagolysosomal system. In contrast, inhibition of the autophagolysosome and proteasome had no effect on CTFs (i.e., C83) and, moreover, impaired the stabilizing effect of DAPT on C83. It is possible that lysosomal inhibition enhances proteasome activity, as well as the activity of other proteolytic systems or the release of exosomes, thereby contributing to C83 elimination, as recently demonstrated [65]. Of particular interest, DAPT-mediated accumulation of C83 was much lower in TgMT5^−/−^ cells than in Tg cells, perhaps reflecting the putative decrease in APPfl revealed by BafA1.

When C99 is overexpressed via the AAV strategy, it is degraded mainly by γ-secretase and to a lesser extent by the proteasome, in contrast to what has been reported in HEK cells [62]. In this context, young neurons could use γ-secretase activity to remove C99, even at the risk of promoting Aβ formation, consistent with the idea that C99 could be more neurotoxic than Aβ [16,17,18,19,66].

## 5. Conclusions

This study confirms the involvement of MT5-MMP in APP metabolism as part of the pathophysiological mechanisms in AD. It also reveals MT5-MMP/IL-1β as a novel CNS neuromodulatory axis that controls neuroinflammation, neuronal activity, and synaptic integrity in developing neurons. Overall, our work highlights the importance of early pathological processes, which may provide new insights into the pathogenesis of AD and the prospects for effective new therapies.

## Figures and Tables

**Table 1:** Intrinsic membrane and spike properties of pyramidal neurons.

		A	B	C	D	E	F	G	H
		WT (n = 7–10)	WT IL-1β (n = 3–15)	MT5^−/−^ (n = 6–15)	MT5^−/−^ IL-1β (n = 5)	Tg (n = 5–10)	Tg IL-1β (n = 5)	TgMT5^−/−^ (n = 6–10)	TgMT5^−/−^ IL-1β (n = 5)
K-Glu	Membrane capacitance (pF)	64.42 ± 12.8	51.2 ± 16.6	50 ± 9.06	71.6 ± 13.8	56 ± 16.87	66.2 ± 22.16	58.43 ± 4.44	54.6 ± 15.11
	Input resistance (MΩ)	194.57 ± 20.2	380.58 ± 53.24	206.75 ± 20.02	216.8 ± 55.75	177.8 ± 35.57	176.8 ± 20.39 B–F ^##^ *p* = 0.003	246.85 ± 47.8	236.4 ± 58.56
CsCl	Membrane capacitance (pF)	65.5 ± 5.95	73.25 ± 20.82	78.13 ± 8.15	84.4 ± 15.13	68.8 ± 9.88	114.2 ± 25.39 E–F ** *p* = 0.0097	73.8 ± 4.95	76.4 ± 11.93
	Input resistance (MΩ)	296.3 ± 40.7	299.18 ± 44.17	354.73 ± 63.70	156.2 ± 28.79	431.6 ± 112.36	166.4 ± 16.32 E–F ** *p* = 0.0261	248.9 ± 30	290.8 ± 136.21
Vrest (mV)		−55.2 ± 2.9	−55.6 ± 7.8	−40.4 ± 2 A–C ^###^ *p* < 0.0001	−54.8 ± 4.8 C–D *** *p* < 0.0001	−55.2 ± 2.3	−48 ± 4.2	−53.9 ± 2.1	−47.8 ± 2.6
V threshold (mV)		−42.1 ± 4.4	ND	−33.9 ± 3.1 A–C ^#^ *p* = 0.037	−26.8 ± 3.4	−38 ± 2.59	−38.2 ± 2.4	−33.2 ± 2	−31.2 ± 2.3
Spike amplitude (mV)		109.7 ± 7.1	ND	85 ± 6.14 A–C ^#^ *p* = 0.024	81.8 ± 5.54	97.6 ± 2.8	95 ± 3.4	100.2 ± 5.09	88.8 ± 6.43
Spike duration (ms)		1.71 ± 0.13	ND	1.87 ± 0.17	2.26 ± 0.19	2.16 ± 0.33	1.76 ± 0.23	2.03 ± 0.14	1.92 ± 0.18

A to H, represents abbreviations for the experimental groups. B-F compares WT IL-β to Tg IL-1β; E-F compares Tg to Tg IL-1 β; A-C compares WT to MT5^−/−^; C-D compares MT5^−/−^ to MT5^−/−^ IL-β. n stands for number of recorded neurons under K-Glu or CsCl conditions, respectively. Note that Vrest, V threshold, Spike amplitude and Spike duration were measured under K-Glu conditions. ** *p* < 0.01 and *** *p* < 0.001 between IL-1β treated and untreated cultures of the same genotype; ^#^ *p* < 0.05 ^##^ *p* < 0.01 and ^###^ *p* < 0.001 comparison between genotypes. ANOVA followed by Fisher’s LSD post hoc test. ND: not determined.

## Data Availability

All data generated or analyzed during this study are included in this published article (and its supplementary additional files) and are available from the corresponding author upon reasonable request.

## Supplementary Materials

The following supporting information can be downloaded at: https://www.mdpi.com/article/10.3390/biom14121645/s1, Figure S1: Expression of MT5-MMP and MT1-MMP in 21 DIV neural cells; Figure S2: Effects of MT5-MMP deficiency on the expression of human *APP*, human *PSEN1* and murine *App* genes; Figure S3: Effects of MT5-MMP deficiency on the expression of murine *Il6*, *Il18* and *Casp1* genes; Figure S4: Effect of MT5-MMP deficiency on N-cadherin levels in 21 DIV neural cells; Figure S5: Representative example of repetitive firing of a WT neuron under untreated conditions; Figure S6: Effects of MT5-MMP deficiency and IL-1β treatment on the expression of genes encoding Aβ-degrading enzymes; Figure S7: Effects of MT5-MMP deficiency, IL-1β and secretase inhibitors on full-length APP levels; Figure S8: Validation of C99 and C83 detection after AAV transduction.
