# Supplementary figures and images for: Suppression of MT5-MMP Reveals Early Modulation of Alzheimer’s Pathogenic Events in Primary Neuronal Cultures of 5xFAD Mice
Source: Biomolecules. 2024 Dec 21;14(12):1645. doi: 10.3390/biom14121645 (PMC11674474; doi:10.3390/biom14121645)

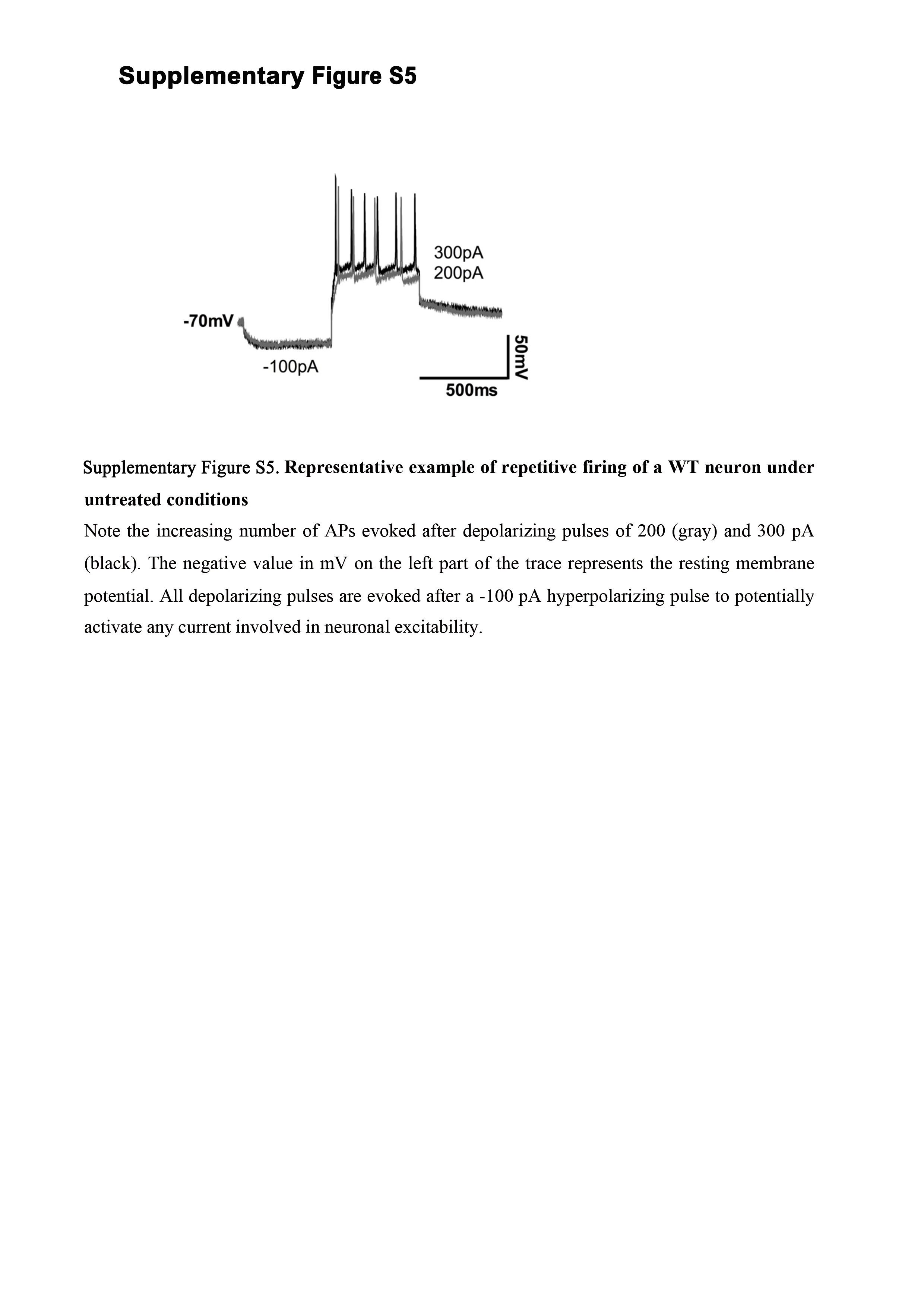

Supplement: Supplementary file 1 [file biomolecules-14-01645-s001.zip › 21_DIV_Supplementary_Figure S5 SR.tif]
